# Assessment of Airway Bronchodilation by Spirometry Compared to Airway Obstruction in Young Children with Asthma

**DOI:** 10.1155/2016/5394876

**Published:** 2016-06-09

**Authors:** Daphna Vilozni, Fahed Hakim, Galit Livnat, Miryam Ofek, Ronen Bar-Yoseph, Lea Bentur

**Affiliations:** ^1^Pediatric Pulmonary Institute, Ruth Rappaport Children's Hospital, Rambam Health Care Campus, 31096 Haifa, Israel; ^2^The Edmond and Lily Safra Children's Hospital, Sheba Medical Center, Sackler Medical School, 52621 Tel Aviv, Israel; ^3^The Bruce Rappaport Faculty of Medicine, Technion-Israel Institute of Technology, 31096 Haifa, Israel

## Abstract

A reversibility test by an increase of greater than 12% in FEV1 can support a diagnosis of asthma and alter a patient's treatment plan but may not be applicable to the young ages. We retrospectively gathered spirometric data from 85/271 asthmatic children having mild obstruction (FEV1 > 80% predicted), age 2.6–6.9 years. Spirometry was performed before and 20 min after inhalation of 200 mcg Albuterol. We defined a deviation below −1.64 *z* scores from control as obstruction and an increased above 1.64 scores from control as a positive response to bronchodilators. Sensitivity of the index was considered significant if it captured >68% of the participants. The sensitivity of detecting airway obstruction in these children by FEV1 was 15.3% and 62.4% by FEF25–75. A positive response to Albuterol was an increase of 9.2% for FEV1 (12% for adults) and 18.5% for FEF25–75. The sensitivity for detecting a response to Albuterol in mild asthma was 64.7% by FEV1 and 91.8% by FEF25–75. Young children having normal spirometry can demonstrate airway reversibility. The response of spirometry parameters to bronchodilators may be more sensitive than obstruction detection and may help to support the diagnosis of asthma and adjust treatment plan.

## 1. Introduction

Asthma is a chronic inflammatory disorder of the airways associated with a variable, widespread, airflow obstruction that is often reversible, either spontaneously or with treatment [1]. A decrease of >20% predicted in FEV1 is considered mild airway obstruction, yet many people with mild asthma may present normal FEV1 values [2]. “Reversibility” is generally applied to rapid improvements of 12% in forced expiratory volume [2, 3] at the first second of expiration (FEV1) measured within minutes after inhalation of a rapid-acting bronchodilator. A reversibility test can support a diagnosis of asthma and can alter a patient's treatment plan and, therefore, it is of clinical importance.

Wheezing, cough, and/or breathlessness are major causes of morbidity in young children regardless of current treatment [4, 5]. Despite recent advances in spirometric measurement techniques, spirometry is rarely used in clinical practice for defining obstruction/reversibility of airways in young children [6, 7]. When spirometry is used, the interpretation of obstruction and a positive response have been based on that observed in adults. The use of FEV1 may be questionable since not all young children will exhale more than one second [6–9]. When exhalation continues beyond one second, the FEV1 value is close to that of the forced expiratory vital capacity (FVC) [6]. FEV0.5 and FEV0.75 have been proposed as surrogates for FEV1 [8, 9]. We reason that there may be differences between the ability to diagnose airway obstruction and dilatation. The aim of this study was to define airway reversibility compared to airway obstruction detection in young children.

## 2. Subjects and Methods

### 2.1. Study Design: Retrospective

 The study design included clinical and spirometry data from children referred to Meyer Children's Hospital during 2011–2014 (data included children from our previous study [9]).

#### 2.1.1. Inclusion Criteria

 Inclusion criteria consisted of data from children who performed spirometry before and 20 min after Albuterol inhalation.

*Asthmatic Children's Data*. Data included children presenting symptoms highly suggestive of asthma according to GINA guidelines [1] and/or frequent episodes of wheezing, activity-induced cough/wheeze, nocturnal cough without viral infections, and absence of seasonal variation. All children had positive challenge tests (either methacholine or exercise).

*Healthy Children's Data*. Healthy children's data included data from children having no recurrent respiratory symptoms or treatment suggestive of asthma, no family history of asthma, and no hospitalization due to respiratory syncytial virus bronchiolitis, with normal baseline spirometry and negative methacholine, who underwent bronchodilator response. Most were evaluated and followed up for habitual cough.

#### 2.1.2. Exclusion Criteria

For asthmatic children, they included chronic respiratory illness other than asthma.

The local ethics committee of Rambam Health Care Campus (Institutional Review Board 0304-11-RambamMC) approved the study.

### 2.2. Methods

Routinely, we instructed children's parents to withdraw Albuterol at least 12 hours before spirometry tests [1].

*Spirometry Tests*. Children performed spirometry in the standing position without a nose clip with a commercial spirometer (KoKo-PDS Dosimeter, nSpire Healthcare Inc., Longmont CO, USA) that includes incentives. Tests were performed according to the official ATS/ERS statement on pulmonary function testing in preschool children [6]. These guidelines include the recommended reproducibility and acceptability to rule out abnormal or unacceptable curves. After satisfactory baseline measurements, children received a bronchodilator (2 puffs of 100 mcg Albuterol) administered via a volumetric spacer. Children repeated the spirometry tests 15–20 min after bronchodilator inhalation.

#### 2.2.1. Data Analysis

We visually inspected each spirometry curve for technical errors and only technically corrected curves were included for analysis. The single best baseline curve values and the best postbronchodilator (BDR) curve values were further analyzed. For predicted values of FVC, FEV1, and FEF25–75, we used the spirometry reference equations from Global Lung Function Initiative [10]. For other indices, we used the predicted values of our former study [11]. When FEV1/FVC > 0.98 was present and the curves were acceptable, we used FEV0.5 as representative of FEV1.

*Obstruction Categories*. We further subgrouped the asthmatic children according to FEV1% predicted values as follows: Mild obstruction included children showing FEV1 ≥ 80% predicted, moderate obstruction included children showing FEV1 of 60–79% predicted, and severe obstruction included children showing FEV1 < 59% predicted. Sensitivity capturing >68% of the participants (values within 1 standard deviation of the mean) was considered clinically significant. We assessed the sensitivity of spirometric indices to detect airway obstruction or dilatation within the three subgroups of asthma severity (see below) and in relation to the healthy population.

*Response to BD*. We defined a meaningful response to bronchodilators by the various spirometry parameters by an elevation of 1.64 *z* scores from the response of our healthy control providing normal distribution.

In capturing either bronchoconstriction or dilatation, a sensitivity >68% (values within 1 standard deviation of the mean) was considered significant.

*Statistics.* Spirometry data was tested for normal distribution. We used paired *t*-tests to compare the differences between baseline lung function and predicted values or differences between prebronchodilator inhalation and postinhalation changes of each subject. Significant differences between the subgroups (healthy, mild, moderate, and severe) were tested by ANOVA. Diagnostic test evaluation was used for sensitivity for each parameter. We used Pearson correlation tests to determine associations between baseline and response to bronchodilators. We report the findings as mean (±SD) when normally distributed or as median and 95% confidence limit if nonnormally distributed. Statistical significance was set at *P* < 0.05.

## 3. Results

We inspected spirometry data from 360 (female, *n* = 157) children. The age composition was as follows: <4.0 years *n* = 90 (17 <3.0 y), between 4 and 5 years *n* = 109, between 5 and 6 years *n* = 102, and between 6 and 7 years *n* = 59. The mean age was 4.8 ± 1.1 years; the mean height for all children was 108 ± 9 cm and weight 19 ± 4 kg.

Table 1 presents the anthropometric data (top table) and baseline spirometry data (bottom table) for healthy and asthmatic children according to subgroups. The table shows that there was no significant difference in the anthropometric data between the groups.

Healthy children's spirometry values (bottom table) did not differ from predicted values for that age group. Healthy children's FEV1/FVC ratio decreased from 0.96 ± 0.01 at age of 3.0–3.9 years to 0.90 ± 0.01 at age of 6.0–6.9 years, similar to reference values. The differences between the two measurements were less than 5% for FVC, FEV0.5, FEV1, and peak flow and less than 9% for FEF50 and FEF25–75; otherwise the curves were not accepted for analysis. FEV1 was unachievable in 46 of the 360 children where FEV0.5 was taken as a substitute.

We found that 85 children had normal to mild obstruction severity, 142 had moderate obstruction, and 44 of the asthmatic group had severe obstruction. FVC decreased in similar magnitude as FEV1 across the different severity's groups, while FEF25–75 decreased significantly more than both FEV1 and FVC (*P* < 0.01) with increasing obstruction severity. FEV1/FVC ratio declined with severity of obstruction from 0.92 ± 0.05 in mild obstruction to 0.87 ± 0.07 in moderate obstruction to 0.83 ± 0.06 in the severe obstruction group (*P* = 0.004).

Table 2 presents the sensitivity of the various spirometry indices to detect either airway obstruction or bronchodilatation according to the subgroups. The sensitivity of detecting airway obstruction in relation to our healthy for the entire asthmatic group was 37.62% for FVC, 45.8 for FEV1, and 75.7% for FEF25–75. The table shows that FEV1 is insensitive to mild and moderate obstruction, while FEF25–75 may capture 62.4% even in mild obstruction and become more sensitive at moderate and severe obstruction.

*Response to Bronchodilators*. The response (% baseline) of healthy controls to bronchodilators was normally distributed and was as follows (mean ± SD): FVC = 0.44 ± 4.33%; FEV1 = 0.53 ± 4.35; FEV0.5 = 0.55 ± 4.3; PEF = 1.09 ± 5.82; FEF25–75 = 3.55 ± 4.71; and FEF50 = 2.60 ± 7.4. A positive response to Albuterol (>1.64 *z*-score from control response) was equal to an increase of 9.7% for FVC, 9.2% for FEV1, and 18.5% for FEF25–75. The 9.2% for FEV1 was lower than the recommended 12% for adults.

Table 2 presents the sensitivity of the various spirometry indices to detect bronchodilatation. The sensitivity for detecting bronchodilation was higher than that of the detection of bronchoconstriction.

**Figure 1:**
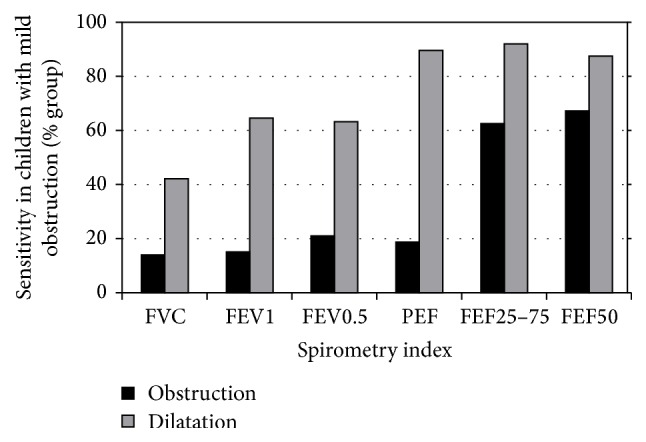
Detection of obstruction and response to BDR by the various spirometry indices, in children with mild obstruction.

Figure 1 presents the detection of obstruction and response to BDR by the various spirometry indices in children with mild obstruction. The figure shows that the sensitivity of mid-flow to BDR is the highest.

**Figure 2:**
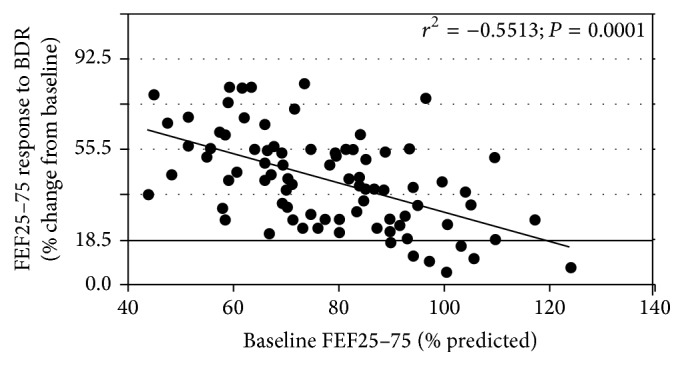
Correlation between airway obstruction severities assessed by FEF25–75 and the response of this index to BDR.

Figure 2 shows the correlation between FEF25–75 values (% predicted) and the response to BDR (% baseline) in children with mild asthma. A significant change in FEF25–75 values in our study was >18.5% from baseline values. It can be seen that the lower the FEF25–75% predicted values, the greater the response to BDR despite the normal FEV1 (*r* ^2^ = −0.5513, *P* = 0.0001).

## 4. Discussion

In this study, we focused on the common spirometry indices to determine the degree of the response to BDR compared to the detection of airway obstruction in young children with asthma of various severities. Our main findings show that rate of detection of response to BDR was higher for all spirometry indices than the rate of detection of bronchial obstruction. The response of FEV0.5, FEF25–75, or FEF50 to bronchodilators captured >68% of the participants, even in children with mild asthma. Conversely, the sensitivity of detection of airway obstruction in mild asthmatics by common parameters (FVC, FEV1, FEV0.5, and PEF) was weak and only mid-flows parameters (FEF25–75/FEF50) captured >68% of this group. Indeed, the response of mid-flow indices correlated with severity of obstruction. These findings may imply that mid-flows are more sensitive in the detection of obstruction and bronchodilation in young asthmatic children.

The definition of airway obstruction and a response to BDR in any studied group depends on defining the response in a healthy group of similar age and gender. Our control group consisted of well-defined, healthy subjects in which asthma was excluded and not a random population. Given the high rate of asthma in the young population, preselecting the control group was more likely to avoid inadvertent inclusion of asthmatic subjects. Our study represents the largest control group evaluated for bronchodilator response in this age group by spirometry. The response of the control group was similar to that published in the literature for various age ranges. Goldstein et al. [12] used the raised flow/volume technique to measure infants aged 0.1–2.9 years and found a response (% from baseline) of 2.2 in FEV0.5 and 3.7 in FEF25–75. Galant et al. tested a population aged 4–17 years and found a change of 2.2% in FEV1 [13]. Dundas et al. [14] assessed healthy children aged 5–10 years in the Dutch CNSLD study group and found a response of 3.8% in FEV1; they also tested an adult population (aged 20–80 years) and found a mean change of 1.0% in FEV1.

The large number included in this study enabled segregation into a spectrum of disease activity. We related the definition of airway obstruction severity to our established reference values [11]. We found that FEV0.5 and FEV1 were similarly low sensitivity indices for detection of mild and moderate airway obstruction. Mid-flows captured more than two-thirds of children with mild obstruction. The use of mid-flow parameters was formerly suggested despite their large standard deviations. Rao et al. [15] studied 437 asthmatic children with normal % predicted FEV1 and found that % predicted FEF25–75 positively correlated with methacholine challenge test results. FEF25–75 also correlated with morning and evening peak expiratory flow [16, 17].

Our study used spirometry to demonstrate airway reversibility in young children. The ability to measure a response to BDR was previously shown by nonspirometric techniques, including force oscillation, specific resistance measured in the plethysmograph [18, 19], and the Rint technique [20]. It is not surprising, therefore, that spirometry could also identify response to BDR in our tested children. In our children, the positive response to Albuterol shown by FEV1 was an elevation of 9.2% and this magnitude of response is lower than recommended for the adult definition of reversibility. Our study suggests that spirometry indices are more sensitive for detecting BDR response than detecting airway obstruction in mild asthma. In response to BDR, FEV0.5 had a sensitivity of similar magnitude to mid-flows. Interestingly, we also found that the change in PEF was similar to that of FEV0.5 in response to BDR. However, FEV0.5 is not mounted on most commercial spirometers and, therefore, we cannot recommend its use.

The sensitivity of an elevation of 18.2% in FEF25–75 was above 91.8%, even in children with mild asthma severity. This positive response correlated with the airway obstruction severity of the entire group. Our findings for higher sensitivity of mid-flow indices are in agreement with previous publications in children [13, 21]. It must be stressed that findings of high sensitivity of mid-flows were unrelated to the changes in FVC, which may result in either elevation or decline in functional residual capacity. We wish to emphasize that spirometry in the preschool age differs from that of the older population, not only in the approach to the child, criteria for acceptability, and repeatability, but also in the shape of the flow-volume of the basic curve. We should also take into consideration the sensitivity of each of the parameters in detecting airway obstruction and bronchodilation.

*Study Limitations*. Ideally, the study would have had a prospective, double-blind design, with at least one repetition on separate days. One could claim that the preschool age is 2–5 years, while we included data from children of up to 6.9 years of age. However, none of the children in this study had entered first grade in school; therefore, they represented preschool ages in our country.

## 5. Conclusion

Our study shows that the sensitivity of spirometric parameters to detect BDR response is significantly higher than the sensitivity to detect obstruction. Lower than recommended magnitude of improvement in FEV1 may be applied in this age group. Peak flow and mid-flows may be considered as sensitive markers for bronchoconstriction and bronchodilation in young children. Bronchodilator response should be assessed in this age group, even when baseline spirometry is within the normal range.

## Figures and Tables

**Table 1:** Anthropometric and baseline lung function (% GLI).

	Healthy (*n* = 89)	Asthma (*n* = 271)		
		Mild (*n* = 85)	Moderate (*n* = 142)	Severe (*n* = 44)
M/F	54/36	50/35	74/68	24/20
Age (y)	5.1 ± 1.1	4.8 ± 0.9	4.7 ± 1.1	4.7 ± 1.1
Ht (cm)	110 ± 8	108 ± 8	109 ± 7	108 ± 9
Wt (kg)	19.5 ± 3.5	19 ± 4	18 ± 4	19 ± 5

Baseline spirometry (% predicted)				
FVC	96 (94–98)	87 (86–91)	72 (72–74)	55 (52–58)
FEV1	102 (100–104)	88 (97–91)	71 (70–72)	55 (51–54)
FEV0.5	98 (98–103)	87 (87–91)	68 (68–71)	57 (53–56)
PEF	92 (90–94)	80 (77–83)	65 (63–68)	48 (49–55)
FEF25–75	91 (90–98)	77 (73–81)	65 (63–70)	48 (43–52)
FEF50	96 (94–101)	71 (67–76)	56 (54–60)	39 (38–44)

Values are presented as median and 95% confidence limit.

**Table 2:** Sensitivity (% population) of spirometric indices for detecting airway obstruction or dilatation.

	Obstruction			Response to BD		
	Mild (*n* = 85)	Moderate (*n* = 142)	Severe (*n* = 44)	Mild	Moderate	Severe
FVC	14.1	40.1	75.0	42.3	64.1	77.3
FEV1	15.3	50.0	90.9	64.7	78.2	86.4
FEV0.5	21.1	56.1	93.1	63.1	70.6	86.4
PEF	18.8	60.5	94.4	89.5	95.1	95.5
FEF25–75	62.4	76.1	97.7	91.8	94.4	93.2
FEF50	67.1	80.0	95.5	87.5	81.2	87.5

## Abbreviations

BDR: Bronchodilators
FEF25–75: Forced expiratory flow between 25% and 75% of vital capacity
FEF50: Forced expiratory flow at 50% vital capacity
FEV1: Forced expiratory volume in 1st second
FEV0.5: Forced expiratory volume in 0.5 seconds
FVC: Forced vital capacity
PEF: Peak expiratory flow.
